# Riding posture affects quadriceps femoris oxygenation during an incremental cycle exercise in cycle‐based athletes

**DOI:** 10.14814/phy2.13832

**Published:** 2018-08-20

**Authors:** Akira Saito, Mitsuki Goda, Takaki Yamagishi, Yasuo Kawakami

**Affiliations:** ^1^ Research Fellow of Japan Society for the Promotion of Science Kojimachi Tokyo Japan; ^2^ Graduate School of Arts and Sciences The University of Tokyo Tokyo Japan; ^3^ School of Sport Sciences Waseda University Saitama Japan; ^4^ Faculty of Sport Sciences Waseda University Saitama Japan

**Keywords:** Cyclists, electromyography, endurance performance, muscle fatigue, near‐infrared spectroscopy

## Abstract

Although oxygenation levels and muscle recruitment patterns of the quadriceps femoris during an incremental cycling exercise has been reported, oxygenation and activation profiles of the quadriceps femoris in racing posture in cycle‐based athletes remain unknown. This study aimed to examine the effects of riding posture on oxygenation and neuromuscular activation of quadriceps femoris during an incremental cycling exercise in cycle‐based athletes. Nine cycle‐based athletes and nine nonathletic subjects performed an incremental cycling exercise at a constant cadence of 90 rpm. Riding postures were the racing posture using an aero‐handle bar (aero posture) and the usual upright racing posture as the control (upright posture). Near‐infrared spectroscopy and surface electromyography were recorded from vastus lateralis and rectus femoris. Changes in the tissue oxygenation index of the near‐infrared spectroscopy from baseline were calculated, and the amplitudes of electromyographic signals were normalized to the initial values of the exercise in each muscle. In cycle‐based athletes, changes in the tissue oxygenation index of vastus lateralis and rectus femoris in the aero posture were significantly lower than those obtained in the upright posture throughout the exercise, whereas no significant differences between the postures were observed in the normalized electromyographic amplitudes of vastus lateralis and rectus femoris. A significant difference between the postures was only occurred in changes of the tissue oxygenation index of rectus femoris in the final phase of exercise in nonathletic subjects. It appears that riding posture affects the oxygenation pattern of quadriceps femoris during incremental cycling exercise in cycle‐based athletes. The main results of this study suggest that aero posture during incremental cycle exercise enhanced the muscular oxygen consumption of the quadriceps femoris in the trained cyclists.

## Introduction

Athletes achieve improvements in their sport performance through musculo‐skeletal, neuromuscular, and cardiovascular adaptations induced by their competitive events and training. In particular, well‐trained cyclists perform a high volume of pedaling exercise training (e.g., professional road cyclists have been reported to cover approximately 30,000 km/year (Hug et al. 2004)) that improves pedaling skill (Cannon et al. 2007), cycling efficiency (Dorel et al. 2009), and muscle energy metabolism (Hug et al. 2006).

Competitive cyclists usually use an aero‐handlebar with the trunk bending forward to minimize their frontal plane area to maximize cycling speed (Grappe et al. 1997). Although trained cyclists could perform similar oxygen consumption between aero‐handlebar and upright postures during an incremental cycle exercise (Origenes et al. 1993), higher oxygen consumption of untrained subjects was obtained in the aero‐handlebar posture than that in the upright posture (Ashe et al. 2003). As the balance between systemic oxygen uptake and muscular oxygen utilization is a major determinant of aerobic exercise performance (Boone et al. 2009; Koga et al. 2014), it seems that physiological determinants of muscular fatigue (e.g., oxygenation status) are affected differently by riding posture during incremental cycling exercise.

Quadriceps femoris (QF) is the primary working muscle during cycling exercise, and it has been reported that riding posture during cycling exercises affects muscle oxygenation (Denis and Perrey 2006; Jones et al. 2006; DiMenna et al. 2010) and activation (Li and Caldwell 1998; Savelberg et al. 2003; Hug and Dorel 2009) of QF. From the functional viewpoint, four QF synergists act as knee extensors, but rectus femoris (RF) also acts as a hip flexor because of its bi‐articular nature. As the QF recruitment patterns have been shown to be related to deoxygenation patterns during a ramp cycle exercise (Chin et al. 2011; Iannetta et al. 2017), trunk position (i.e., hip joint angle) and changes in the length of RF, changing riding posture may induce alterations of oxygenation and muscle activation of RF during cycling. However, the QF oxygenation and activation profiles in the racing posture in cycle‐based athletes remain unknown.

Accordingly, this study examined the effect of riding posture on oxygenation and neuromuscular activation of QF during an incremental cycling exercise in cycle‐based athletes. The aim was to test the hypothesis that less muscle oxygenation saturation and greater neuromuscular activation would be observed in RF during the racing posture in the cycle‐based athletes. Moreover, in an attempt to determine the effects of pedaling skills on oxygenation and neuromuscular activation, measurements of nonathletic subjects were also performed.

## Materials and Methods

### Subjects

Nine cycle‐based athletes (seven males and two females) and nine nonathletic subjects (seven males and two females) participated in this study. The mean ± standard deviation age, height, and weight of the athletes were 21.3 ± 1.5 years, 169.0 ± 5.7 cm, and 59.8 ± 7.7 kg, respectively, and those of the nonathletic subjects were 26.0 ± 4.7 years, 167.8 ± 8.7 cm, and 63.5 ± 11.0 kg, respectively. The nine cycle‐based athletes included seven triathletes and two cyclists. They had a minimum of 2 years of training and racing experience, and they were in preparation for a full competitive season. The nonathletic subjects varied in physical activity level ranging from untrained to regularly physical active, but they did not report a cycling training history. None of them had recent or chronic cardiovascular or metabolic diseases or pathology of the limb muscles or joints. Each subject was informed of the purpose, procedures, and possible risks of the measurements of this study and provided written, informed consent to be involved in this study. The study was approved by the Institutional Ethical Committee on Human Research, and carried out in line with the Declaration of Helsinki.

### Experimental protocol

**Figure 1:**
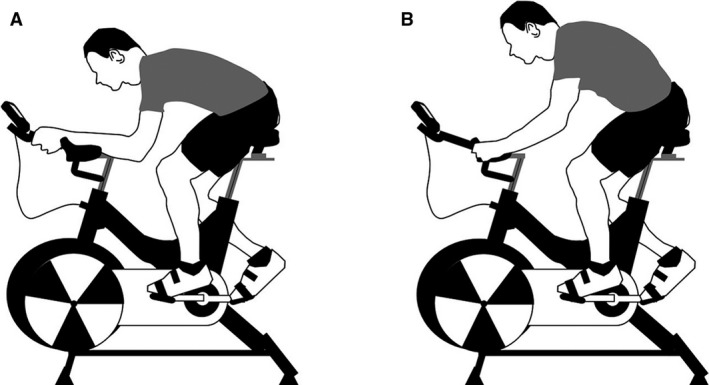
Illustrations of riding posture during cycling exercise. An incremental test was performed in the aero posture (A) and the upright posture (B). (A): Subjects put their forearm on the aero‐handle bar. (B): Subjects gripped the bracket part of the handle‐bar.

All experimental protocols were carried out in our laboratory, where temperature was maintained at 23°C. Subjects visited the laboratory on two separate occasions over a 2‐day period to perform an incremental cycling test on air and magnetic‐braked cycle ergometer (Wattbike Pro, Wattbike Ltd., UK). Measurements of trained cyclists using this ergometer have shown high reliability for their power output (Hopker et al. 2010) and attenuation of pedaling asymmetry (Kell and Greer 2017). All subjects performed the measurements in two riding postures: one measurement was performed in the racing posture using an aero‐handle bar (i.e., aero posture; Fig. 1A), and the other was in the usual racing posture (i.e., upright posture; Fig. 1B). The ergometer was equipped with a standard crank (length = 170 mm) and saddle height was adjusted to ensure a knee joint angle of 170° at the bottom of the crank (Saito et al. 2015). The handlebar height was set to match the comfortable position of each subject and the feet were strapped to the pedals. Each subject completed a 5‐min warm‐up protocol at constant moderate intensity ranging from 100 to 150 W. After a 2‐min resting period on the cycle ergometer, an incremental test started at 130 W, and then the work rate was increased by 25 W every minute. Subjects were instructed to maintain a crank cadence of 90 rpm. Tests were terminated when the subjects could not maintain a cadence beyond 85 rpm for at least 15 sec despite strong verbal encouragement by examiners. The measurement at each posture was separated by at least 24 h, and the order was randomized.

### Near‐infrared spectroscopy recording

Muscle oxygenation of vastus lateralis (VL) and RF of the right leg during cycling was continuously monitored using a multichannel near‐infrared spectroscopy (NIRS) apparatus (NIRO‐200, Hamamatsu Photonics, Japan). The NIRS apparatus provided a derived tissue oxygenation index (TOI; [HbO_2_]/[HbO_2_ + HHb] × 100), indicating the mean saturation of the hemoglobin volume present within the microvasculature. [HbO_2_] and [HHb] represent oxyhemoglobin and deoxyhemoglobin, respectively. The TOI may serve as a better estimate of muscle fractional O_2_ extraction than [HHb] (Kime et al. 2013), and it is insensitive to changes in total microvascular hemoglobin content compared to [HHb] (Quaresima and Ferrari 2009). In fact, changes of VL blood volume was sensitive to the crank angle within a cycle and crank cadence (Takaishi et al. 2002). Moreover, TOI of VL was highly reproducible during a constant load cycling exercise (Niemeijer et al. 2017). Therefore, the TOI was the main parameter in this study.

The optodes were housed in a plastic holder (A9782, Hamamatsu Photonics). Prior to attaching the optodes, the skin was shaved, abraded, and cleaned with alcohol. The optode for VL was placed at the mid‐point between the head of the greater trochanter and the inferior edge of the patella. The optode for RF was attached at the mid‐point of the line joining the anterior superior iliac spine and the patella. The optodes were secured on the skin surface with double‐sided adhesive tape (A9342, Hamamatsu Photonics) in order to minimize the loss of infrared light and intrusion of extraneous light from outside the field of interrogation. The NIRS data were sampled at 6 Hz and synchronously digitized with EMG data on a personal computer using specific software (Chart 7, ADInstruments, Australia).

### Surface electromyography recording

Surface electromyographic (EMG) signals during cycling were recorded from VL and RF of the right leg using a wireless EMG system (Trigno, Delsys). The EMG electrodes for VL and RF were placed parallel to each optode. Two silver bar electrodes, with an inter‐electrode distance of 10‐mm, were used for EMG acquisition from each of these muscles. The EMG signals were recorded in differential derivation, with a bandpass filter at 20–450 Hz and sampled at 1000 Hz using an AD converter (PowerLab, ADInstruments) synchronized with the NIRS data on the computer using Chart 7 software (ADInstruments).

### Data analysis

The mean TOI of the NIRS and the root‐mean‐square (RMS) of the EMG signals in VL and RF were continuously calculated every second. The second‐by‐second TOI and RMS data were smoothed by a five‐point moving average (Chin et al. 2011). Change in the TOI of each muscle from baseline until the exhaustion was expressed as ΔTOI (Hopker et al. 2017). The baseline was defined as the mean TOI value measured over the final 30 sec of the rest period (DiMenna et al. 2010). The smoothed RMS of each muscle was normalized to the RMS value recorded during the initial 1 min of the incremental test. Change in the normalized RMS of each muscle was expressed as ΔRMS. These data were plotted as percentages of the exercise duration and divided every 10% of the duration.

### Statistics

The exercise durations and peak power output during the incremental cycling tests in the aero and upright postures were analyzed using two‐way (posture × group) analysis of variance (ANOVA) with repeated measures. When a significant difference in the duration between the groups was found, an individual Student's *t*‐test was performed to determine differences between the groups in each posture. The ΔTOI and ΔRMS of VL and RF were analyzed using two‐way (time × posture) ANOVA with repeated measures. Greenhouse‐Geisser corrections were used when sphericity was violated. In the case of a significant interaction or main effect, a paired *t*‐test was performed to compare between the postures. The level of statistical significance was set at *P* < 0.05. Statistical analyzes were performed using SPSS statistics version 24 (IBM, Japan).

## Results

The exercise durations in the aero and upright postures in cycle‐based athletes were 7.9 ± 1.8 min and 8.1 ± 2.0 min, respectively. Those in nonathletic subjects were 5.5 ± 1.5 min and 6.0 ± 1.7 min, respectively. Cycle‐based athletes could perform the incremental exercise significantly longer than noncyclists in both postures (*P* = 0.014). Neither of the groups showed a significant difference in duration between the postures (*P* = 0.056). The peak power output in the aero and upright postures in cycle‐based athletes were 296.6 ± 43.3 W and 302.2 ± 53.6 W, respectively. Those in nonathletic subjects were 235.5 ± 37.0 W and 249.4 ± 41.0 W, respectively. The peak power output in cycle‐based athletes was significantly higher than nonathletic subjects (*P* = 0.012). No significant difference in the peak power output between the postures in the both groups (*P* = 0.094).

### Oxygenation of quadriceps femoris

**Figure 2:**
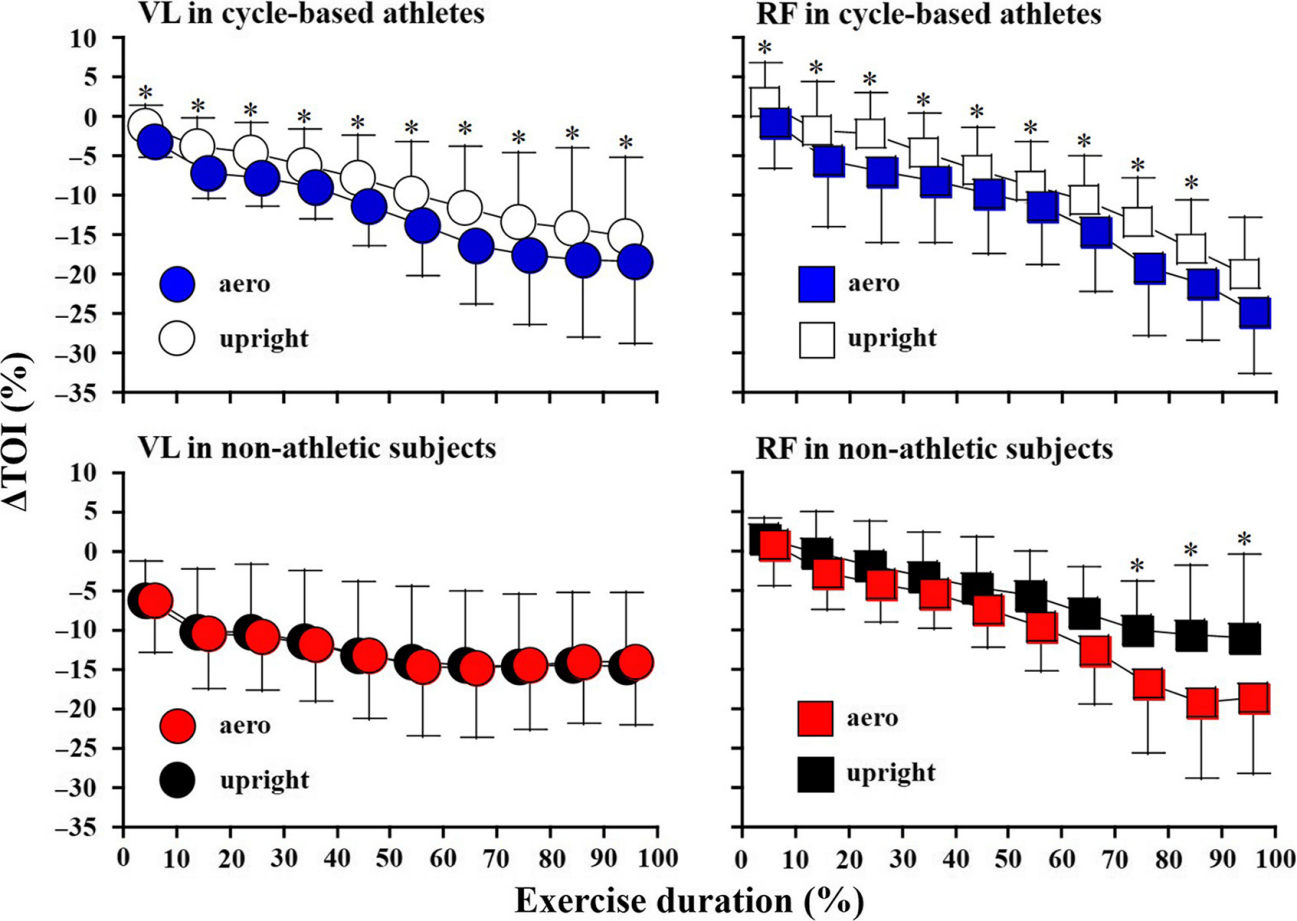
Tissue oxygenation index of quadriceps femoris during an incremental cycling test. All data were expressed by mean and standard deviation. The figures indicate the oxygenation of vastus lateralis (VL) and rectus femoris (RF) in the cycle‐based athletes and nonathletic subjects, respectively. *P < 0.05, aero posture versus upright posture.

While no significant time‐by‐posture interaction effect (VL: *P* = 0.603; RF: *P* = 0.526) was confirmed, significant main effects of posture and time were found in the ΔTOI of VL and RF in the cycle‐based athletes (VL: *P* = 0.003; RF: *P* = 0.003) (Fig. 2). ΔTOIs of VL and RF in the aero posture were significantly lower than in the upright posture (*P* = 0.009‐0.031), except for that of RF at 90–100% of exercise duration (*P* = 0.127). In the nonathletic subjects, no significant time‐by‐posture interaction (*P* = 0.770) and posture (*P* = 0.963) effects were observed in the ΔTOI of VL, whereas there was a significant main effect of time (*P* = 0.005). There was a significant time‐by‐posture interaction effect in the ΔTOI of RF in the nonathletic subjects (*P* = 0.010). At 70–100% of the duration, ΔTOI of RF in the aero posture was significantly lower than that in the upright posture (*P* = 0.002–0.025).

### Neuromuscular activation of quadriceps femoris

**Figure 3:**
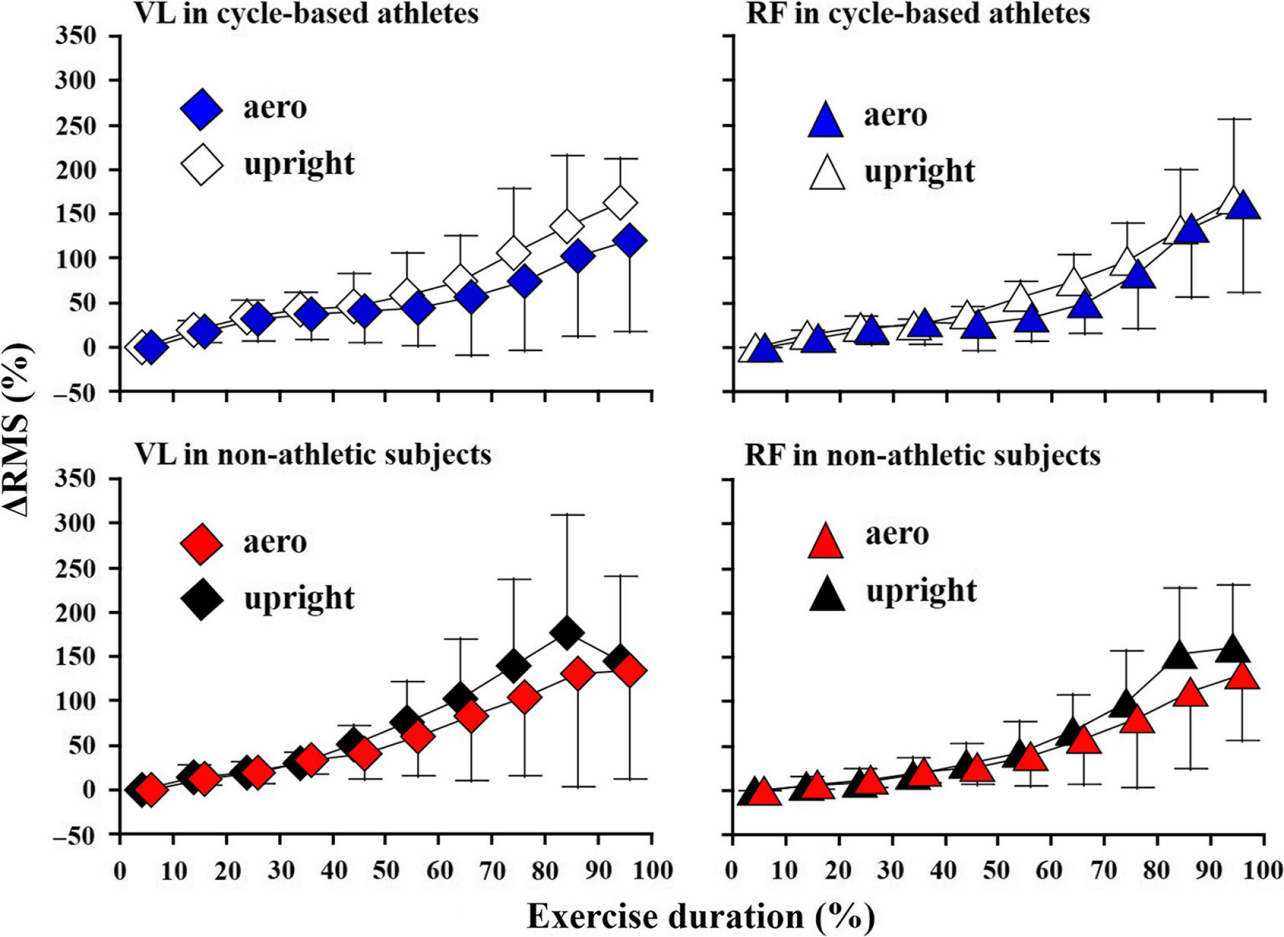
Neuromuscular activation of quadriceps femoris during an incremental cycling test. All data were expressed by mean and standard deviation. The figures indicate the activation of vastus lateralis (VL) and rectus femoris (RF) in cycle‐based athletes and nonathletic subjects, respectively.

There was no significant time‐by‐posture interaction (VL: *P* = 0.289; RF: *P* = 0.684) or posture effect (VL: *P* = 0.283; RF: *P* = 0.603) in the ΔRMS of VL and RF, whereas the time effects of those of VL and RF were significant in the cycle‐based athletes (VL: *P* = 0.012; RF: *P* = 0.003) (Fig. 3). Likewise, the nonathletic subjects showed no significant time‐by‐posture interaction (VL: *P* = 0.308; RF: *P* = 0.544) or posture effect (VL: *P* = 0.374; RF: *P* = 0.794) in the ΔRMS of VL and RF, but the time effects of those of VL and RF were significant (VL: *P* = 0.006; RF: *P *< 0.001).

## Discussion

The effect of riding posture on oxygenation and neuromuscular activation of QF was examined during an incremental cycling exercise. The main findings of this study were that (1) QF muscle tissue oxygen saturation in the aero posture was lower than that in the upright posture throughout the exercise, and (2) the neuromuscular activation pattern was similar between the postures in the cycle‐based athletes. These findings partly supported our hypothesis that less muscle oxygen saturation and greater neuromuscular activation would be observed in RF in the aero posture.

It has been reported that posture has an effect on muscle deoxygenation of VL in noncyclists during high‐intensity cycling exercise (Denis and Perrey 2006; DiMenna et al. 2010). Denis and Perrey (2006) showed that VL deoxygenation was greater in the supine than in the upright posture throughout a high‐intensity, constant‐load cycling exercise in noncyclists, despite its mono‐articular nature. Their findings are in line with another previous study that used an incremental ramp cycling exercise (DiMenna et al. 2010). Furthermore, they have reported the presence of postural effect (supine vs. upright) on maximal power output during incremental cycling (Denis and Perrey 2006; DiMenna et al. 2010). However, postural effect on ΔTOI of VL and maximal power output was not occurred in the nonathletic subjects in the current study. In the final phases of the exercise, the RF TOI in the aero posture was less than that in the upright posture in the nonathletic subjects (Fig. 2). Furthermore, the effect of riding posture on the ΔTOI during incremental cycling was observed in RF only, suggesting that muscle length influences muscle tissue saturation of RF. Thus, in an exhausted condition, the change in muscle length may affect the balance between O_2_ delivery and O_2_ utilization within the working muscle.

As opposed to the nonathletic subjects, the greater TOI changes in VL and RF observed in the aero posture in the cycle‐based athletes (Fig. 2). Charlton et al. (2017) showed that work and power of breathing in aero posture increased than those in upright posture, and less efficient ventilation response to cycle exercise was observed in the aero posture in male cyclists. Since greater mechanical cost of breathing during aero posture cycling led to ventilatory inefficiency (Charlton et al. 2017), postural effect on the muscular oxygenation saturation of VL and RF was obtained in cycle‐based athletes.

Aerobic fitness of the subjects is one of factors explaining the difference in QF oxygenation patterns profiles (Boone et al. 2009; Okushima et al. 2016). With respect to this point, a previous study showed that significant relationship between VO_2peak_ and tissue oxygen saturation of VL and RF during incremental ramp cycle exercise (Okushima et al. 2016). In addition, Boone et al. (2009) examined deoxygenation patterns of VL in trained cyclists and physically active students during a ramp cycling exercise and demonstrated that the level of aerobic fitness affects the dynamics of deoxygenation of VL. Although maximal aerobic power (i.e., *V*O_2max_) was not measured in this study, the difference in time to exhaustion (i.e., cycle‐based athletes > nonathletic subjects) between the groups indicates that the cycle‐based athletes may have had greater aerobic capacity than their nonathletic counterparts. Since endurance training increases muscle oxidative capacity (Gollnick and Saltin 1982) and the amount of slow twitch fibers (Harber et al. 2012), the differences in oxidative capacity and/or muscle fiber type distribution between the subjects may be potential factors that influence QF oxygenation.

As a primary factor causing the different postural effects on VL oxygenation patterns during the cycling exercise, a previous study implied that more VL motor units are recruited in the supine posture with increasing cycling workload than in the upright position (DiMenna et al. 2010). In this study, while the findings obtained from the cycle‐based athletes showed postural effects on muscle oxygenation saturation of VL and RF, postural effects on neuromuscular activation were not confirmed in either muscle (Fig. 3). Similar QF recruitment patterns between the postures in both groups may reflect that neural control of QF during cycling may be independent of the trunk position (Hug et al. 2011; Verma et al. 2016). Hug et al. (2011) examined the EMG signals of 11 lower limb muscles in well‐trained athletes during seated and standing all‐out sprint cycling, and they then extracted muscle synergies using a non‐negative matrix factorization algorithm. They found that three muscle synergies accounted for the majority of variabilities in 11 muscle activation patterns, and these three synergies were not sensitive to a change in posture (Hug et al. 2011). Furthermore, consistent with the present observations, the increase pattern of EMG activity in VL was similar between supine and upright postures during high‐intensity constant‐load cycling (Denis and Perrey 2006). Therefore, we assume that the neural control strategy of QF during incremental cycling is fairly constant across different riding postures.

There was a methodological limitation in this study which should be taken into consideration. Physiological characteristics of the subjects were not sufficient to classify both subject groups. This study did not measure pulmonary oxygen uptake for each subject during incremental cycle exercise. If this study examined the relationship between gross oxygen uptake and oxygen utilization of the QF during the cycling exercise, underlying physiological mechanism inducing the postural effect on the QF oxygenation in cycle‐based athletes could have been revealed.

## Conclusion

Oxygenation and neuromuscular activation of QF were examined during an incremental cycling exercise with two different riding postures. Muscle tissue oxygen saturation of VL and RF in the aero posture was lower than that in the upright posture throughout the exercise in the cycle‐based athletes, whereas the neuromuscular activation pattern was similar between the postures in these athletes. In the nonathletic subjects, the postural effect on muscle oxygenation was observed in RF only in the final phases of the exercise. Neuromuscular activation of VL and RF was similar between the postures throughout the exercise in both groups. The main results of this study suggest that bending forward posture during incremental cycle exercise enhanced muscular oxygen consumption of the quadriceps femoris in cycle‐based athletes.

## Conflict of Interest

All authors have reported no relevant conflicts of interest.
